# Recurrent KRAS, KIT and SF3B1 mutations in melanoma of the female genital tract

**DOI:** 10.1186/s12885-021-08427-x

**Published:** 2021-06-08

**Authors:** Yuan-jun Cai, Long-feng Ke, Wen-wen Zhang, Jian-ping Lu, Yan-ping Chen

**Affiliations:** 1 Department of Obstetrics and Gynecology, Fujian Maternity and Child Health Hospital, Affiliated Hospital of Fujian Medical University, Fuzhou, 350001 China; 2 Laboratory of Molecular Pathology, Fujian Medical University Cancer Hospital, Fujian Cancer Hospital, Fuzhou, 350014 China; 3 Department of Pathology, Fujian Medical University Cancer Hospital and Fujian Cancer Hospital, No 420, Fuma Road, Fuzhou, 350014 Fujian Province China

**Keywords:** Malignant melanoma, Female genital tract, Mutation, Targeted therapy, Driver genes

## Abstract

**Background:**

Malignant melanoma of the female genital tract is relatively uncommon and accounts for 3–7% of all melanoma localizations. This study aimed to identify driver genes in melanoma of the female genital tract with the purpose of enhancing understanding of disease pathogenesis and identifying potential new therapeutic targets to develop effective therapies.

**Methods:**

KIT (CD117) and BRAF expression were detected immunohistochemically. Polymerase Chain Reaction (PCR) and Sanger sequencing techniques were performed to identify the mutational status of *BRAF, NRAS, KRAS, NF1, KIT, PDGFRA* and *SF3B1* on 19 melanomas of the female genital tract, paired with 25 cutaneous melanomas, 18 acral melanomas and 11 melanomas of nasal cavity.

**Results:**

Somatic variant analysis identified *KRAS* (6/19; 32%) as the most commonly mutated gene, followed by *KIT* (4/19; 21%), *SF3B1* (3/19; 16%) and *NRAS* (1/19; 5%). None of the cases were found to harbor *BRAF, NF1* and *PDGFRA* mutations in melanomas of the female genital tract. However, none of the cases were found to harbor *SF3B1* and *KIT* mutations in cutaneous melanomas, acral melanomas and melanomas of nasal cavity. Recurrent *KIT* mutations, as well as mutations in the less frequently mutated genes *NRAS* and *SF3B1,* were exclusively detected in vulvovaginal melanomas, but not in tumors arising in the cervix. However, recurrent *KRAS* mutations were detected in similar frequencies in tumors of the vulva, vagina, and cervix. Additionally, recurrent *KRAS* and *KIT* mutations occurred predominantly in polygonal and epithelioid cell types of melanoma in the female genital tract. Immunohistochemistry revealed moderate or strong cytoplasmic CD117 expression in 6 of the 19 cases (31.6%).

**Conclusions:**

We observed that gynecologic melanoma harbored distinct mutation rates in the *KIT, BRAF, SF3B1, KRAS, and NRAS* genes. Our findings support the notion that gynecologic melanoma is a distinct entity from non-gynecologic melanoma, and these findings offer insights into future therapeutic options for these patients.

**Supplementary Information:**

The online version contains supplementary material available at 10.1186/s12885-021-08427-x.

## Introduction

Primary malignant melanomas arise from melanocytes which present in the genital mucosal epithelium of the female genital tract are extremely rare, accounting for 3–7% of all melanoma localizations [1, 2]. The most common site of melanomas of the female genital tract is the vulva (75%), followed by the vagina, and 20% of cases are multifocal [3]. Other sites such as cervix, uterus and ovary are very rare. Patients more frequently present with early metastatic spread and poor prognosis, and the 5-year overall survival rate is approximately 10% [4]. In recent years, many of the advances made in the treatment of cutaneous melanoma with the aim of improving overall survival, but not readily applicable to mucosal melanomas [5, 6].

Mucosal melanomas are not related to sun exposure or other known environmental factors; therefore, mucosal melanomas have been shown to have distinct molecular profiles compared with cutaneous melanoma arising on sun-exposed sites [7, 8]. Some common activating driver mutations in genes identified in cutaneous melanoma, such as mutated *BRAF V600E* [9], have less frequently identified in mucosal melanoma [10–19]. To better understand the mutational profile and identify potential new therapeutic targets of mucosal melanoma, several investigators have performed next-generation sequencing on a small number of mucosal melanomas from different anatomical sites and compared the results with cutaneous melanoma [7, 20–23]. Hayward and colleagues [21] found that acral and mucosal melanomas were dominated by structural changes and mutation signatures of unknown etiology that were not previously identified in melanoma, such as in *SF3B1*. Newell and colleageues [22] also found that mucosal melanomas had a low point mutation burden and high numbers of structural variants; the significantly mutated genes in mucosal melanomas included *NRAS, BRAF, NF1, KIT, SF3B1, TP53, SPRED1, ATRX, HLA-A* and *CHD8* genes. These studies have provided the foundation for understanding the molecular profiles of mucosal melanoma and expanded our knowledge of this rare disease. The distinct mutation profiles of cutaneous and mucosal melanoma are striking and support a model of different developmental pathways.

To better understand the mutational profile and provide important new clues into the molecular changes that occur in melanomas of the female genital tract, our study analyzed the molecular drivers of 19 melanomas of the female genital tract (vulva, vagina and cervix), paired with 25 cutaneous melanomas, 18 acral melanomas and 11 melanomas of nasal cavity. Our findings expand this knowledge by contributing the large cohort of mucosal melanoma of the female genital tract known to date with validated mutations and may lead to a better understanding of the molecular drivers, and hence improved therapeutics for these rare forms of melanoma.

## Material and methods

### Case collection and histological assessment

Nineteen melanomas of the female genital tract, 25 cutaneous melanomas, 18 acral melanomas and 11 melanomas of nasal cavity were obtained from the case files of the Department of Pathology of Fujian Cancer Hospital, China, from October 2010 to September 2019. The biopsy material was fixed in 10% formalin and embedded in paraffin after routine histological tissue processing. Formalin-fixed paraffin-embedded tissue (FFPE) sections (3–4 μm thick) were stained with hematoxylin-eosin for microscopic examination. All cases were carefully reviewed independently by two melanoma pathologists to confirm the histological diagnosis according to the criteria of the 2014 WHO Classification of Tumors Female Reproductive Organ, and extensive review of medical records was performed. This study was carried out in accordance with the Declaration of Helsinki and written informed consent was obtained from the patients or their legal guardians. The study protocol was approved by the institutional review boards of Fujian Cancer Hospital.

### Immunohistochemistry

Immunohistochemistry assays were performed on diagnosed patient tissues available in the form of FFPE tissue blocks using Bond-III Autostainer (Leica Biosystems, Melbourne, Australia) in the Department of Pathology’s clinical immunohistochemistry laboratory. Immunostaining on paraffin sections was done for CK, EMA, S-100, HMB45, Melan-A, SOX-10, Ki67, KIT (CD117) and BRAF (using antibody clones from Maixin Biotech Co., Ltd., Fuzhou China). Conditions for individual immunohistochemistry assays including antigen retrieval and antibody dilutions varied according to the antibody used and were determined by standard optimization and validation procedures. Positive and negative staining controls were included as appropriate.

### DNA isolation

Five pieces (5-μm-thick sections) were cut from FFPE tumor tissues. The sections were deparaffinized and manually microdissected according to standard procedures. Genomic DNA was isolated using the QIAamp FFPE DNA Tissue Kit (Qiagen, Germantown, MD, USA) according to the manufacturer’s instructions. Samples were quantified using the Qubit DNA high sensitivity assay (Life Technologies, Carlsbad, USA).

### Sanger sequencing

Nested PCR was performed to amplify *BRAF* exon 15; *NRAS* exons 2, 3 and 4; *KRAS* exons 2, 3 and 4; *KIT* exons 9, 11, 13, 17 and 18; *PDGFRA* exons 12 and 18; *NF1* exon 22; and *SF3B1* exon 14 on 19 melanomas of the female genital tract, as previously described [24–26]. PCR was carried out using AmpliTaq Gold polymerase (Applied Biosystems, Weiterstadt, Germany) according to the manufacturer’s instructions under the following conditions: 95 °C for 5 min followed by 38 cycles of denaturation for 30 s at 94 °C, annealing for 30 s at 58 °C and extension for 60 s at 72 °C. The primer pairs for *BRAF*, *NRAS*, *KRAS*, *PDGFRA*, *KIT*, *NF1* and *SF3B1* were designed using Primer Premier 5. The PCR products were routinely purified and sequenced in both directions using the BigDye Terminator version 3.1 Cycle Sequencing Kit (Applied Biosystems, Foster City, CA, USA). At least two independent PCR and sequencing experiments were conducted to confirm mutations.

### Next-generation sequencing assay

The panel of targeted genes was designed on the basis of large-scale mutation-profiling studies on melanomas covering *BRAF, NRAS, KRAS, NF1, KIT, PDGFRA* and *SF3B1* [21, 22]. The genomes of 25 cutaneous melanomas, 18 acral melanomas and 11 melanomas of nasal cavity with eligible formalin-fixed and paraffin-embedded samples were sequenced using Nextseq. 500 (Illumina, Inc., USA) instrument. The raw data were aligned and analyzed for the detection of insertions/deletions and single-nucleotide variants. The detail process of NGS library preparation, capture-based targeted DNA sequencing and data analysis were described in supplementary material.

## Results

### Clinicopathologic findings of patients with melanomas of the female genital tract

This study included 19 patients with primary melanomas of the female genital tract, with a median patient age of 53.0 years and an average age of 55.4 years at diagnosis (range from 34 to 84 years). The primary melanomas of the female genital tract included five vulvar (26.3%), eight vaginal (42.1%) and six cervical melanomas (31.6%). Six (31.6%) patients had T4 disease, two (10.5%) had T3 disease, three (15.8%) patients had T1 disease and the remaining eight (42.1%) patients had T2 disease. The clinicopathological features of the patients are summarized in Table 1.

**Table 1:** Cumulative clinical data and mutational status for 19 patients with mucosal melanoma of female genital tract

	Clinical information				Mutational status							CD117 expression
Patients	Tumor location	Age at diagnosis	Clinical stage	histological subtype	*SF3B1*	*KIT*	*PDGFRA*	*BRAF*	*NRAS*	*KRAS*	*NF1*	
P01	vagina	56	I	spindle	–	–	–	–	–	–	–	
P02	vagina	40	II	mixed type	–	–	–	–	–	–	–	
P03	vulva	50	III	mixed type	–	–	–	–	p.Q61 *	–	–	
P04	vagina	53	II	epithelioid	–	p.S476N p.G498V	–	–	–	–	–	+
P05	vulva	53	I	epithelioid	–	p.L640P	–	–	–	–	–	+
P06	vagina	77	II	epithelioid	–	–	–	–	–	–	–	
P07	cervix	48	I	epithelioid	–	–	–	–	–	–	–	
P08	vagina	84	II	epithelioid	–	–	–	–	–	–	–	
P09	vagina	52	II	epithelioid	–	p.D810H	–	–	–	p.G13D	–	+
P10	cervix	42	IV	spindle	–	–	–	–	–	p.G13D	–	
P11	vulva	59	II	epithelioid	p.R625H	–	–	–	–	p.G13D	–	
P12	cervix	55	IV	epithelioid	–	–	–	–	–	p.G12D	–	+
P13	vulva	74	IV	mixed type	–	–	–	–	–	–	–	
P14	vulva	72	IV	spindle	p.R625H	–	–	–	–	–	–	
P15	vagina	56	IV	epithelioid	–	–	–	–	–	p.G12D	–	
P16	cervix	42	II	spindle	–	–	–	–	–	–	–	
P17	cervix	34	III	mixed type	–	–	–	–	–	–	–	
P18	vagina	52	II	epithelioid	p.R625H	p.V852A	–	–	–	–	–	+
P 19	cervix	54	IV	epithelioid	–	–	–	–	–	p.G13D	–	+

the variant type: SNV; mutation type: missense_variant; mixed type: both epithelioid and spindle tumor cells

**Fig. 1:**
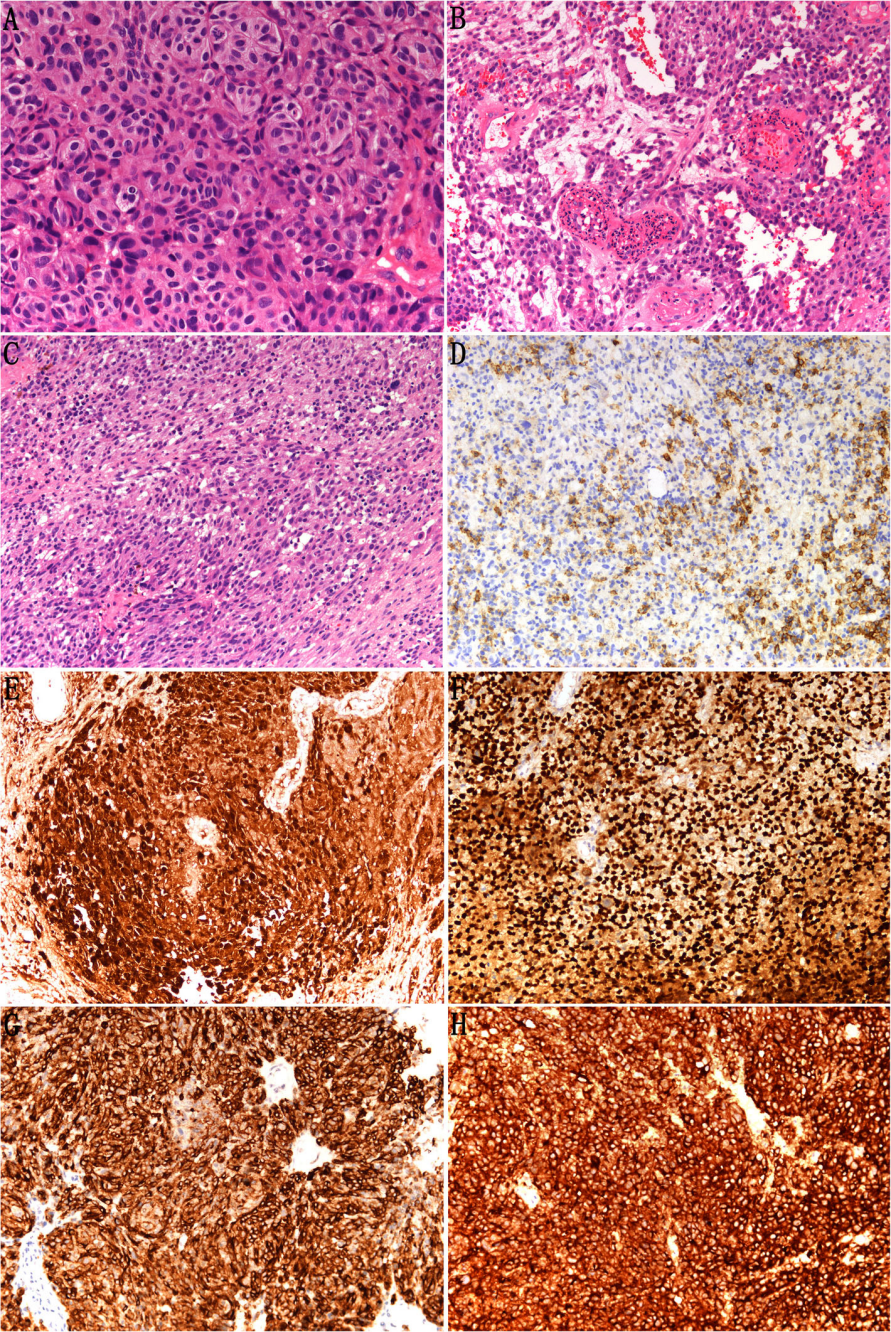
Representative hematoxylin and eosin staining and immunostaining of mucosal melanoma of the female genital tract. Microscopic examination revealed that the tumor cells were distributed in solid islets, nests, or band-like formations. Tumor cells were polygonal (A), epithelioid (B), or spindle in shape (C) (× 200 magnification). Immunohistochemical analysis revealed that CK expression was negative in most cases, but weakly focally positive in three of the 19 cases (D) (× 200 magnification). Tumor cells were strongly positive for Melan-A (E), HMB45 (F), S100 (G) and SOX-10 (H) protein in vulvar malignant melanomas (× 200 magnification)

Microscopic examination revealed that the tumor cells were distributed in solid islets, nests or band-like formations. Tumor cells were polygonal, epithelioid or spindle in shape and rarely of small cell type, with oval, pleomorphic, hyperchromic nuclei (Fig. 1A–C). In most cases, diffuse or focal tumor cells contained dark-brown intracellular melanin pigment. No melanin pigment was found microscopically in a few cases. Mitoses were easy to see. The average number of mitotic figures in the tumor cells per square millimeter was 3–5. Immunohistochemical analysis revealed that CK was negative in most cases and weakly focally positive in three cases (3/19, 15.8%) (Fig. 1D and Table 1). The tumor cells were strongly positive for S-100 and SOX-10 protein (Fig. 1 E–F). Positive Melan-A and HMB-45 protein expressions were also detected, with somewhat lower intensity of staining (Fig.1 G–H). Tumor cells were negative for EMA (data not shown). These findings supported the diagnosis of melanoma.

### CD117 and BRAF expression in patients with melanomas of the female genital tract

**Fig. 2:**
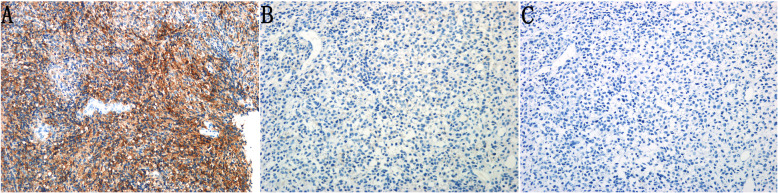
Representative immunostaining of CD117 and BRAF in mucosal melanomas of the female genital tract. (A) Representative staining showing tumor cells strongly positive for CD117. (B) Representative staining showing tumor cells negative for CD117. (C) Representative staining showing tumor cells negative for BRAF

Immunohistochemistry revealed moderate or strong cytoplasmic CD117 expression in 6 of the 19 cases (31.6%) (as shown in Fig. 2A). The remaining cases (68.4%) were negative for CD117 (as shown in Fig. 2B). Interestingly, none of the cases were positive for BRAF (as shown in Fig. 2C).

### BRAF, NRAS, KRAS, NF1, KIT, PDGFRA and SF3B1 gene mutation analysis

**Fig. 3:**
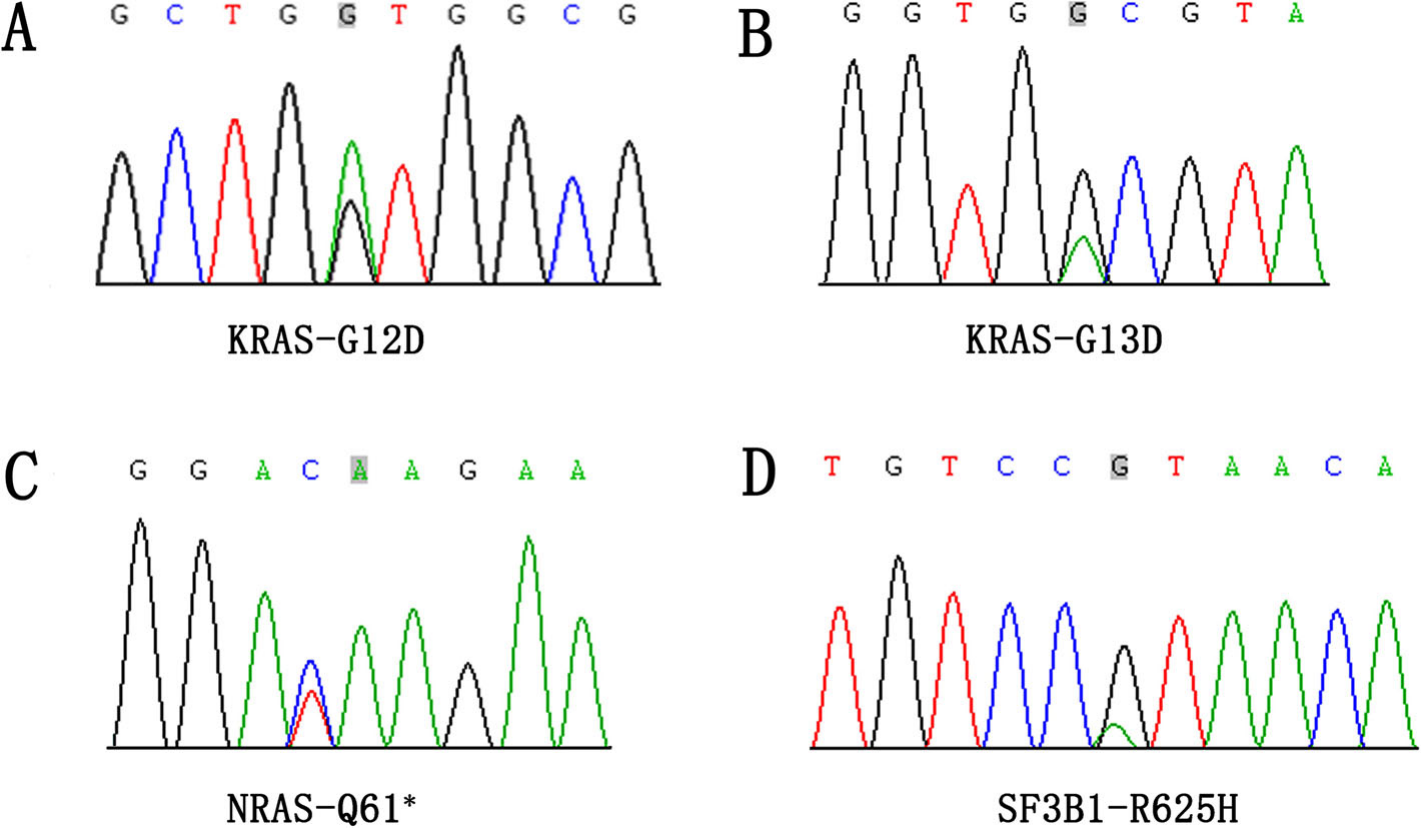
Representative examples of *KRAS* (A, G12D; B, G13D), *NRAS* (C, Q61*) and *SF3B1* (D, R625H) mutations identified in mucosal melanoma of the female genital tract

**Fig. 4:**
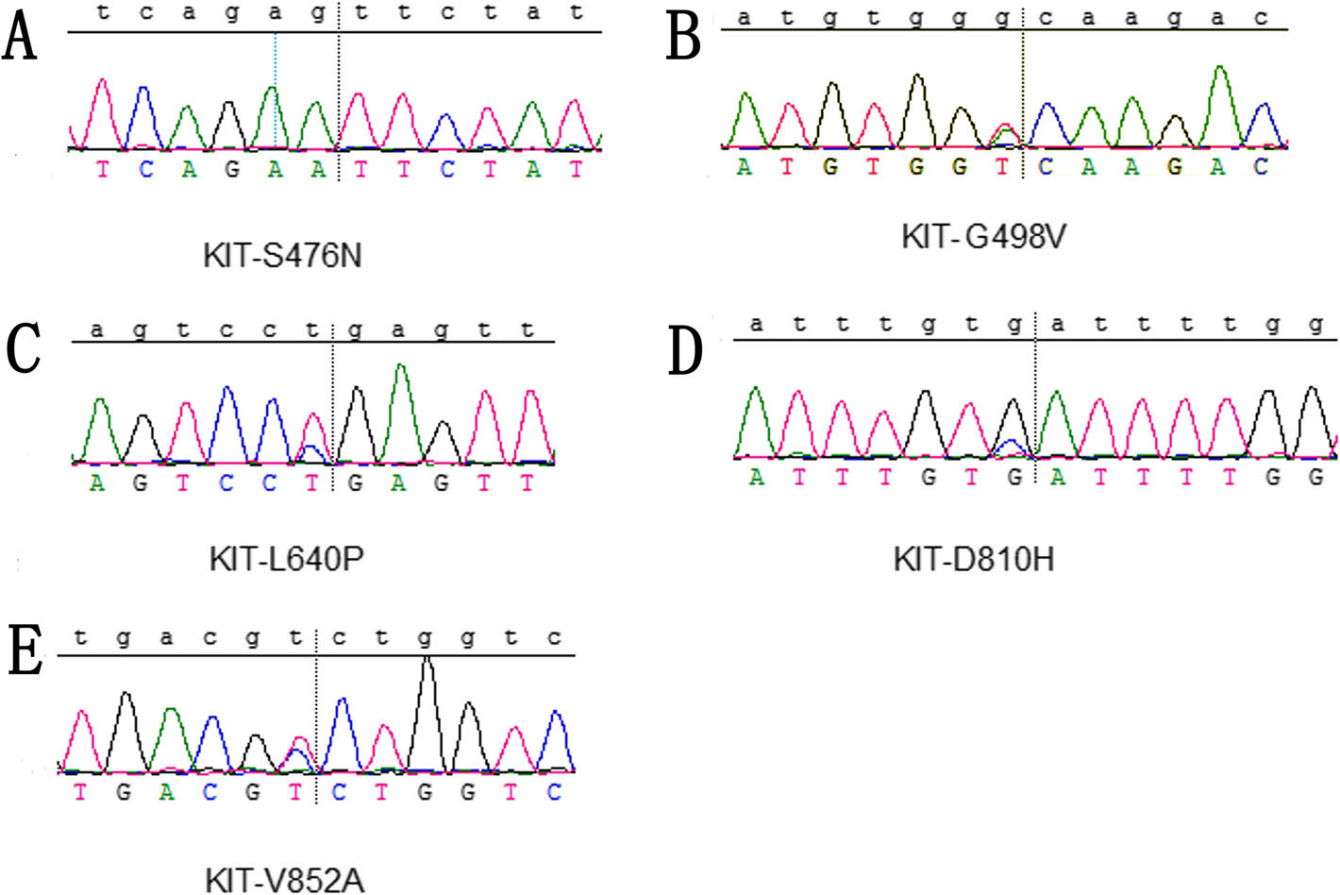
Representative examples of *KIT* (A, S476N; B, G498V; C, L640P; D, D810H; E. V852A) mutations identified in mucosal melanoma of the female genital tract

We examined gene mutations in the 19 melanomas of the female genital tract (listed in Table 1). We identified six cases with *KRAS* mutations (four p.G13D, two p.G12D) and one cases with *NRAS* mutation (p.Q61*) (Fig. 3 A–C). In total, 37% of tumors showed either a *KRAS* or *NRAS* mutation (32% *KRAS* mutation, 5% *NRAS* mutation). The *SF3B1* p.R625H hotspot mutation was detected in 16% (3/19) of the mucosal melanomas of the female genital tract (Fig. 3D). Notably, recurrent *KIT* mutations (p.S476N, p.G498V, p.L640P, p.D810H, p.V852A) were found in 21% (4/19) of the melanomas of the female genital tract; one sample was found to harbor both p.S476N and p.G498V mutations (Fig. 4). In addition, one case had both *KRAS* and *C-KIT* mutations. All four tumors with recurrent *KIT* mutations showed strong CD117 immunostaining. In contrast, oncogenic driver mutations in BRAF, which are commonly identified in cutaneous melanoma, were not detected in any sample (data not shown). None of the cases were found to harbor *NF1* and *PDGFRA* mutations (data not shown).

Notably, recurrent *KIT* mutations, as well as less frequent gene mutations in *NRAS* and *SF3B1*, were exclusively detected in vulvovaginal melanomas, but not in tumors arising in the cervix (Table 1). However, recurrent *KRAS* mutations were detected at a similar frequency in tumors of the vulva, vagina and cervix. Additionally, high numbers of *KRAS* and *KIT* mutations were identified with frequencies varying according to histological subtype. Interestingly, the recurrent *KRAS* and *KIT* mutations occurred predominantly in polygonal and epithelioid cell subtypes, but rarely in spindle cells, in melanoma of the female genital tract.

We next examined gene mutations in 25 cutaneous melanomas, 18 acral melanomas and 11 melanomas of nasal cavity (listed in Supplementary Table 1). We identified *BRAF V600E* (11/25; 44%) as the most commonly mutated gene, followed by *NRAS* (2/25; 8%), and *KRAS* (1/25; 4%) in cutaneous melanomas. While, *BRAF V600E* mutation *was* less frequent in acral melanomas (1/18;6%). In total, 28% (3/18) of acral melanomas showed either a *KRAS* or *NRAS* mutation (11% *KRAS* mutation, 17% *NRAS* mutation). Interestingly, the *BRAF V600E* hotspot mutation was not detected in the melanomas of nasal cavity. We identified five cases with *NRAS* mutations (5/11;45%) and two cases with *KRAS* mutations (2/11;18%) in the melanomas of nasal cavity. Notably, none of the cases were found to harbor *SF3B1, NF1, KIT* and *PDGFRA* mutations in cutaneous melanomas, acral melanomas and melanomas of nasal cavity. In a word, compared with gynecologic melanoma, non-gynecologic melanoma harbored distinct mutation rates in *KIT, BRAF, SF3B1, KRAS* and *NRAS* genes.

## Discussion

Primary malignant melanomas of the female genital tract are extremely rare. The clinical behavior and molecular characteristics of these melanomas have not been well explored. Although melanoma of the female genital tract is an aggressive disease with histological resemblance to melanomas of other sites, recent studies found the heterogeneity of molecular biology of melanoma of different sites. Up to date, relatively little information is known about the molecular alterations that drive melanoma of the female genital tract [10–18]. To better understand the mutational profile and offer insights into future therapeutic options for patients with melanomas of the female genital tract, our study analyzed the histological and genetic characteristics of 19 melanomas of the female genital tract (vulva, vagina and cervix), paired with 25 cutaneous melanomas, 18 acral melanomas and 11 melanomas of nasal cavity.

Activating V600E or V600K mutations in BRAF kinase have been observed in up to 62% of melanomas arising in sun-exposed skin. However, in melanomas arising on mucosal surfaces or non-sun-exposed skin, BRAF mutations are infrequently reported [11]. Previous studies showed that BRAF was mutated in 0 to 33% of patients with vulvar and vaginal melanomas with sample sizes ranging from 1 to 51 cases [27–29]. In our study, oncogenic driver mutations in *BRAF V600E*, which were commonly identified in 44% cutaneous melanoma, were not detected in the melanomas of female genital tract. Our finding is similar to most published data on vulvovaginal melanomas [10–18]. The differences between our findings and some published studies reporting on BRAF mutations in urogenital melanomas or vulvovaginal melanomas are unclear. We doubt the small number of samples in our series (19 patients) could account for this discrepancy. One explanation may lie in the use of different mutation screening methods, which vary in sensitivity. In our study, Sanger sequencing (covering exon 15) was used to detect *BRAF* mutation. In contrast, Hou and colleagues [27] used a combination of next-generation sequencing (covering exons 1–18) and Sanger sequencing (covering exons 11 and 15). In addition, many of their samples were metastatic and may have harbored mutations that differed from the molecular makeup of the primary tumor. Notably, only some of the BRAF-mutant vulvar and vaginal melanomas in the literature harbored BRAF V600E mutations [27–29]. A literature search of the remaining BRAF variant-mutant including BRAF G469A, D594E, D594N, D594H in vulvovaginal melanomas revealed possible inactivating mutations that were less likely to respond to vemurafenib, which is the FDA-approved selective inhibitor of the V600E mutant BRAF kinase used to treat patients who have metastatic or unresectable melanoma with BRAF mutations [27]. Our results indicate that none of the patients with melanomas of the female genital tract can be treated with vemurafenib.

According to the literatures, *KRAS* mutations are common in pancreas, colon and lung cancers [30], whereas *NRAS* mutations are common in myeloid leukemias and cutaneous melanomas [30–32]. However, we identified six *KRAS* mutations and one *NRAS* mutation in 19 melanomas of the female genital tract. In total, 37% of tumors showed either a *KRAS* or *NRAS* mutation (32% *KRAS*, 5% *NRAS*). As reported previously, the mutations were found to be mutually exclusive. In our study, the prevalence of *KRAS* mutation in melanomas of the female genital tract was notably higher than melanomas of other sites, whereas the prevalence of *NRAS* mutation in melanomas of the female genital tract was notably lower compared with the prevalence in melanomas arising in nasal cavity, where mutation rates of up to 45%. Our finding is similar to the published data on esophageal melanomas, which harbored *NRAS* mutations in 30% of cases [33]. Recurrent *KRAS* or *NRAS* mutation contribute to poor prognosis [30–32]. However, in recent years, MEK inhibition was shown to demonstrate therapeutic activity in NRAS-mutated melanoma in clinical trials, opening a novel therapeutic era for these tumors [34].

*KIT* mutations have been observed in varying frequencies in melanomas arising at different primary sites [18, 20]. KIT protein expression or overexpression as detected by immunohistochemistry has been reported to show some correlation with *KIT* gene mutations but has been insufficient to predict response to KIT-targeted therapy with imatinib [16, 18]. In our study, moderate or strong cytoplasmic KIT expression was detected in 6 of the 19 cases (31.6%), and *KIT* mutations were observed in 21% (4/19) of the mucosal melanomas of female genital tract. All four tumors with *KIT* mutations showed strong KIT immunostaining. This finding shows that KIT protein expression correlated with *KIT* mutation. The frequency of *KIT* mutation in our series was much higher than rates reported in studies on non-gynecologic melanoma [27]. Interestingly, *KIT* mutations were associated with histological subtype and tumor site. Notably, recurrent *KIT* mutations were exclusively detected in vulvovaginal melanomas, but not in tumors arising in the cervix, and *KIT* mutation varied immensely between vulvar and vaginal sites, with 20% (1/5) of vulvar samples harboring the mutation compared with only 37.5% (3/8) of vaginal samples. This further highlights our conclusion that mucosal melanomas of the female genital tract have a genetic profile that is distinct from that of mucosal melanomas from different anatomical sites. In addition, we found that *KIT* mutations occurred predominantly in polygonal and epithelioid cell subtypes, but rarely in spindle cells. However, our findings are different from those of Hou and colleagues [27] that showed that vulvar melanoma may be associated with a much higher *KIT* mutation rate than vaginal melanoma. The differences between our findings and published studies on *KIT* mutations in vulvar and vaginal melanomas could be due to the small numbers of samples in our series, different methodology or ethnic difference. It is also possible that vulvar tumors were regarded as melanomas of non-sun-exposed areas. In addition, co-mutations of KIT and NF1 have been reported in mucosal melanoma, although they are rare [35]. However, in our study, none of the cases were found to harbor *NF1* and *PDGFRA* mutations in melanomas of the female genital tract, as well as in cutaneous melanomas, acral melanomas and melanomas of nasal cavity. I think the small number of samples in our series could account for this discrepancy.

*SF3B1* mutations have been identified in subsets of solid tumors, as well as in myelodysplastic syndrome and chronic lymphocytic leukemia [36–38]. Recently, *SF3B1* was identified as a significantly mutated gene in mucosal melanoma, especially in uveal, female genital and anorectal melanomas [20–23]. Our study also found that the *SF3B1* R625 hotspot mutation occurred in 16% of the mucosal melanomas of the female genital tract and not detected in cutaneous melanomas, acral melanomas and melanomas of nasal cavity. *SF3B1* mutations have different prognostic associations in different types of cancers. In uveal melanoma, *SF3B1* mutations are associated with a better prognosis, whereas in other mucosal melanomas [36], *SF3B1* mutations are correlated with a worse prognosis [25]. However, the study of these rare tumors was underpowered to detect statistically significant differences, and larger studies are required to address this issue. This finding suggests that SF3B1 might be exploited as a novel prognostic and/or therapeutic target in melanomas of the female genital tract.

## Conclusion

We observed that gynecologic melanoma harbored distinct mutation rates in *c-KIT, BRAF, SF3B1, KRAS* and *NRAS* genes compared with non-gynecologic melanoma. Our findings support the notion that gynecologic melanoma is a distinct entity from non-gynecologic melanoma, especially cutaneous melanomas. Although our results are preliminary, they highlight the unique molecular landscape of gynecologic melanoma within the spectrum of melanoma malignancies, and these findings offer insights into future therapeutic options for these patients.

## Supplementary Information

**Additional file 1.**

## Data Availability

The data used to support the findings of this study are available from the corresponding author by request.
