# Supplementary material for: Recurrent KRAS, KIT and SF3B1 mutations in melanoma of the female genital tract
Source: BMC Cancer. 2021 Jun 8;21:677. doi: 10.1186/s12885-021-08427-x (PMC8185938; doi:10.1186/s12885-021-08427-x)
Supplement: Supplementary file 1 — Additional file 1. [file 12885_2021_8427_MOESM1_ESM.docx]

# Recurrent KRAS, KIT and SF3B1 mutations in melanoma of the female genital tract

**Running head: Genetic features of the melanoma of the female genital tract**

Yuan-jun Cai^1#^, Long-feng Ke^3#^, Wen-wen Zhang^2^, Jian-ping Lu^2^, Yan-ping Chen ^2*^

^1^ Department of Obstetrics and Gynecology, Fujian Provincial Maternity and Children’s Hospital, Affiliated Hospital of Fujian Medical University, Fuzhou 350001, China;

^2^ Department of Pathology, Fujian Medical University Cancer Hospital and Fujian Cancer Hospital, Fuzhou 350014, China;

^3^ Laboratory of Molecular Pathology of Fujian Cancer Hospital, Fujian Medical University Cancer Hospital, Fuzhou, 350014, China.

Yuan-jun Cai and Long-feng Ke contributed equally to this work as the co-first authors.

Correspondence: Yan-ping Chen

Department of Pathology of Fujian Medical University Cancer Hospital and Fujian Cancer Hospital, No 420, Fuma Road, Fuzhou, Fujian Province, 350014, China.

Tel 86+0591-83660063

Fax 86+0591-62752890

Email [kelf2006@126.com](mailto:kelf2006@126.com)

1. **NGS library preparation, sequencing and data analysis**

At least 50ng of DNA is required for NGS library. Tissue DNA was sheared using Covaris M220, followed by end repair, phosphorylation, and adaptor ligation. Fragments of size 200–400bp were selected by AMPure beads (Agencourt AMPure XP Kit), followed by hybridization with capture probe baits, hybrid selection with magnetic beads and PCR amplification. The quality and size of the fragments were assessed using a bioanalyzer high-sensitivity DNA assay. Indexed samples were sequenced on Nextseq500 sequencer (Illumina, Inc., USA) with pair-end reads. Sequence data were mapped to the human genome (hg19) using BWA aligner 0.7.10. Local alignment optimization, variant calling and annotation were performed using GATK 3.2, MuTect, and VarScan. Variants were filtered using the VarScan fpfilter pipeline, with loci with depth less than 100 filtered out. Base calling in tissue samples required at least 5 and 8 supporting reads for small insertion-deletions (INDELs) and single nucleotide variants (SNVs), respectively. According to the ExAC, 1000 Genomes, dbSNP, ESP6500SI-V2 database, variants with population frequency over 0.1% were grouped as SNP and excluded from further analysis. The mean read depth resulting from the sequencing is 500×.

1. **The panel of Next-Generation Sequencing in our study.**

| AKT1 | NRAS | CDA | GSTP1 | IDH2 |
| --- | --- | --- | --- | --- |
| BRAF | PDGFRA | CYP19A1 | MDR1 | TERT |
| HRAS | PIK3CA | CYP2C19 | MTHFR | SMAD4 |
| KIT | SMO | CYP2C8 | NQO1 | NOTCH1 |
| KRAS | TSC1 | CYP2C9 | RRM1 | IDH1 |
| EGFR | ALK | CYP2D6 | GGH |  |
| FGFR1 | ROS1 | DHFR | UGT1A1 |  |
| FGFR2 | RET | DPYD | XRCC1 |  |
| HER2 | NTRK1/2/3 | ERCC1 | SLC19A1 |  |
| MET | SF3B1 | ERCC2 | NF1 |  |

**3. Table 1. The variant type, mutation type and allele frequency of all identified mutations of cutaneous melanomas,** **acral melanomas and melanomas of nasal cavity**

| **Name** | **gene** | **var_type** | **mutation_type** | **description** | **af** |
| --- | --- | --- | --- | --- | --- |
| Cutaneous melanomas |  |  |  |  |  |
| Case01 | BRAF | SNV | missense_variant | p. V600E | 55.91% |
| Case02 | BRAF | SNV | missense_variant | p. V600E | 8.25% |
| Case03 | BRAF | SNV | missense_variant | p. V600E | 51.18% |
| Case04 | BRAF | SNV | missense_variant | p. V600E | 34.42% |
| Case05 | BRAF | SNV | missense_variant | p. V600E | 54.87% |
| Case06 | NRAS | SNV | missense_variant | p.Q61K | 52.95% |
| Case07 | BRAF | SNV | missense_variant | p. V600E | 53.10% |
| Case08 | BRAF | SNV | missense_variant | p. V600E | 24.52% |
| Case09 | BRAF | SNV | missense_variant | p. V600E | 30.61% |
| Case10 | KRAS | SNV | missense_variant | p. G12D | 2.02% |
| Case11 | BRAF | SNV | missense_variant | p. V600E | 19.46% |
| Case12 | BRAF | SNV | missense_variant | p. V600E | 45.05% |
| Case13 | NRAS | SNV | missense_variant | p. Q61R | 43.30% |
| Case14 | BRAF | SNV | missense_variant | p. V600E | 14.88% |
| Acral melanomas |  |  |  |  |  |
| Case01 | BRAF | SNV | missense_variant | p. V600E | 23.41% |
| Case02 | KRAS | SNV | missense_variant | p. G12D | 16.91% |
| Case03 | NRAS | SNV | missense_variant | p. Q61R | 32.62% |
| Case04 | KRAS | SNV | missense_variant | p. G13D | 20.58% |
| Case05 | NARS | SNV | missense_variant | p. G12D | 57.46% |
| Case06 | NARS | SNV | missense_variant | p. Q61R | 65.72% |
| Melanomas of nasal cavity |  |  |  |  |  |
| Case01 | NRAS | SNV | missense_variant | p. G12D | 70.96% |
| Case02 | NRAS | SNV | missense_variant | p. Q61K | 29.62% |
| Case03 | KRAS | SNV | missense_variant | p. Q61R | 60.81% |
| Case04 | KRAS | SNV | missense_variant | p. G12V | 19.47% |
| Case05 | NRAS | SNV | missense_variant | p. Q61R | 17.82% |
| Case06 | NRAS | SNV | missense_variant | p. G13R | 6.43% |
| Case07 | NRAS | SNV | missense_variant | p. G12D | 79.76% |

**4. Figure. 1** **The positive and negative control of CD117 and BRAF.**


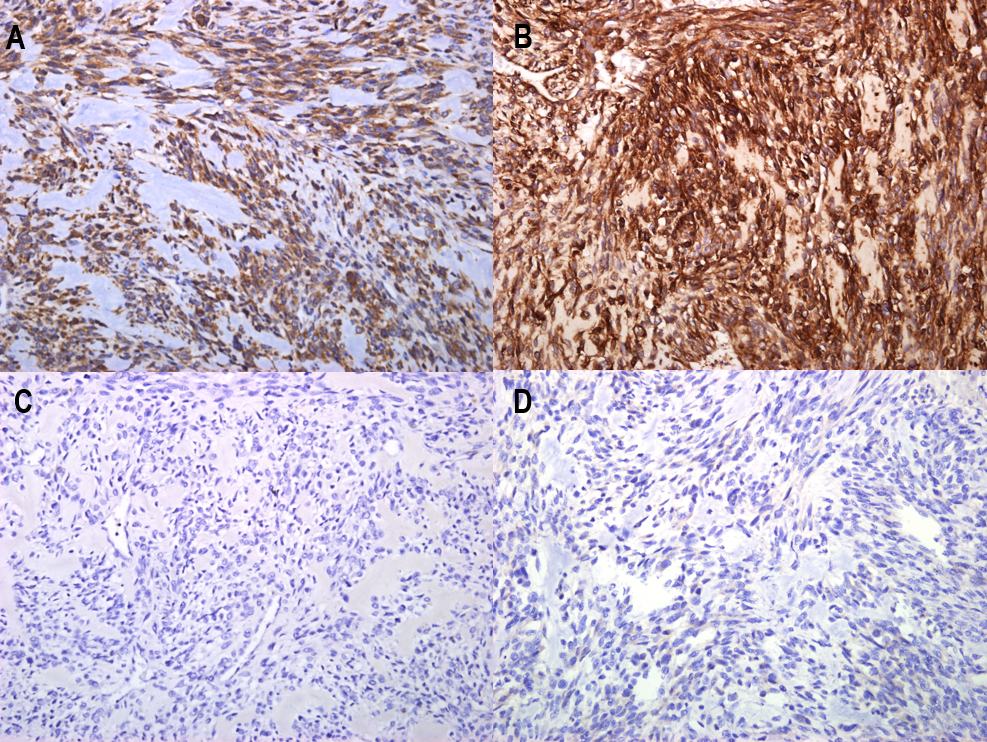


**A. The positive control of BRAF.**

**B. The positive control of CD117.**

**C. The negative control of BRAF.**

**D. The negative control of CD117.**
